# Spatial heterogeneity of the association between fine particulate matter and lung cancer incidence in Hebei Province

**DOI:** 10.3389/fonc.2026.1780882

**Published:** 2026-03-23

**Authors:** Ze Liu, Chang Yan, Zhiqiang Yan, Xin Su, Zheng Li, Yan-Yu Liu, Yutong He

**Affiliations:** 1 Department of Thoracic Surgery, The Fourth Hospital of Hebei Medical University, Shijiazhuang, China; 2 School of Public Health, Hebei Medical University, Shijiazhuang, China; 3 Cancer Institute in Hebei Province, The Fourth Hospital of Hebei Medical University, Shijiazhuang, China

**Keywords:** geographically weighted regression, Hebei Province, lung cancer, PM_2.5_, spatial heterogeneity

## Abstract

**Background:**

Long-term exposure to particulate matter (PM_2.5_) can increase the population’s risk of lung cancer incidence. This study aims to further analyze the spatial heterogeneity in the association between PM_2.5_ and lung cancer incidence.

**Methods:**

This study utilized lung cancer incidence data (2015–2022), PM_2.5_ concentrations, and other covariates from ten cities in Hebei Province. By comparing the Ordinary Least Squares (OLS) and Geographically Weighted Regression (GWR) models, it aimed to comprehensively assess the spatial heterogeneity in the association between PM_2.5_ and lung cancer incidence.

**Results:**

For every 10 μg/m^3^ increase in PM_2.5_ concentration, the overall relative risk (RR) of lung cancer incidence in Hebei Province was 1.079 (95% CI: 1.054–1.104). The RR associated with each 10 μg/m³ increment in PM_2.5_ varied geographically, ranging from 1.026 to 1.086 across the study area, demonstrating a spatial pattern characterized by higher RR values in the north and lower values in the south. Notably, in regions with lower baseline PM_2.5_ concentrations, the same incremental increase in PM_2.5_ (10 μg/m³) was associated with a greater elevation in lung cancer risk.

**Conclusion:**

The association between PM_2.5_ and lung cancer incidence varies spatially across cities in Hebei Province. This suggests that, while continuing to emphasize air quality improvement in heavily polluted areas, governments and administrative authorities should also address regions with lower pollution levels. Tailored air quality management strategies and targeted allocation of healthcare resources should be implemented according to local conditions.

## 1 Introduction

Lung cancer constitutes a major threat to human life and health. Globally, it remains the leading cause of both cancer incidence and mortality (1). According to the 2022 cancer statistics released by the National Cancer Center of China, lung cancer also ranks first in both the incidence and mortality of malignant tumors among both men and women in China (2), imposing a considerable disease burden that demands full attention.

Air pollution is the second leading cause of lung cancer, which includes particulate matter (PM) and gaseous pollutants. PM is the mixture of solid and liquid particles grouped by aerodynamic diameter. Gaseous pollutants that are potential health hazards include ground level ozone (O_3_), volatile organic compounds (VOCs), sulfur dioxide (SO_2_) and nitrogen oxides (NOx). Among them, the most harmful to respiratory health is particulate matter with aerodynamic diameter less than 2.5 microns (PM_2.5_) (3). PM_2.5_ is characterized by its small particle size, large specific surface area, and strong adsorption capacity. These properties enable it to carry toxic substances such as heavy metals, carcinogenic organic compounds, and bacteria. Upon inhalation, these particles can penetrate deep into and remain within the respiratory and cardiovascular systems, potentially inducing cardiopulmonary diseases. Pre-existing pulmonary conditions may subsequently progress or contribute to the development of lung cancer (4). Extensive epidemiological and toxicological evidence has established that long-term exposure to PM_2.5_ is associated with an increased risk of lung cancer incidence and mortality (5). In October 2013, the International Agency for Research on Cancer (IARC) classified PM_2.5_, a key component of outdoor air pollution, as a Group 1 carcinogen to humans (6). The early 21st century witnessed rapid urbanization and industrialization in China, which resulted in severe air pollution and heightened public concern over the health effects of PM_2.5_ (7). In Northern China, particularly the Beijing-Tianjin-Hebei region, concentrated industrial activity, heavy reliance on coal, and dense vehicular emissions have led to exceptionally high PM_2.5_ concentrations, making it one of the most polluted areas in the country.

While numerous epidemiological studies in China have established a long-term association between PM_2.5_ exposure and lung cancer mortality (8, 9), research focusing on incidence remains limited. Given that disease incidence represents the initial health outcome of exposure and is less confounded by disparities in healthcare access and treatment efficacy, investigating the PM_2.5_-lung cancer incidence relationship is of critical public health importance. Owing to its vast territory and uneven socioeconomic development, China exhibits substantial regional variations in PM_2.5_ concentration and composition. Consequently, a growing body of evidence suggests that the association between PM_2.5_ and lung cancer also exhibit significant geographical heterogeneity (10, 11). The Geographically Weighted Regression (GWR) model is a key spatial analytical tool in environmental health studies, as it quantifies how the association between variables changes across geographical space (12, 13). Identifying the magnitude and spatial pattern of this variation is crucial for pinpointing regions where environmental risk factors exert a stronger influence. Moreover, existing research suggests that the effect of PM_2.5_ exposure on lung cancer is not only subject to a time lag (14) but also demonstrates significant geographic variation in its strength (15).

Therefore, this study utilized lung cancer incidence data from 2015 to 2022 across ten cities in Hebei Province (excluding Langfang). The cross-correlation function (CCF) was employed to determine the optimal lag period for the PM_2.5_-lung cancer association. The Ordinary Least Squares (OLS) and Geographically Weighted Regression (GWR) model was subsequently applied to comprehensively analyze the spatial heterogeneity of this association. The findings aim to provide a scientific basis for implementing differentiated air pollution control measures and optimizing the allocation of healthcare resources in Hebei Province.

## 2 Materials and methods

### 2.1 Study area

Located in the North China region, Hebei Province covers a total area of 188,800 square kilometers and administers 11 cities. By the end of 2022, it had a resident population of 74.2 million, with an urbanization rate of 61.65%. The province is characterized by a temperate continental monsoon climate. The vector map of Hebei Province used in this study was obtained from the Hebei Provincial Geographic Information Public Service Platform (https://hebei.tianditu.gov.cn/bzdt/), with the map review approval number of Ji S (2025) 009.

### 2.2 Research data

#### 2.2.1 Lung cancer incidence

Lung cancer incidence data for Hebei Province from 2015 to 2022 were obtained from the Hebei Provincial Office for Cancer Prevention and Control. Case identification and classification followed the International Classification of Diseases, 10th Revision (ICD-10), with cases coded C33-C34 being selected for this study. Corresponding population data were sourced from the local public security or statistical bureaus associated with each registry.

By 2022, Hebei Province maintained 47 cancer registries, covering ten prefecture-level cities (excluding Langfang). The combined population covered by these registry areas was 27,237,886, accounting for approximately 36.71% of the province’s total resident population at the end of 2022. All data collection, collation, and analysis were performed by specialized personnel, ensuring high data quality that is representative of the malignant tumor burden in Hebei Province. The study protocol was reviewed and approved by the Medical Ethics Committee of the Fourth Hospital of Hebei Medical University (Approval No. 2025KS111).

#### 2.2.2 PM_2.5_ exposure

The PM_2.5_ exposure data utilized in this study were obtained from the China High-Resolution High-Quality PM_2.5_ Dataset (https://doi.org/10.5281/zenodo.3539349), hosted by the National Tibetan Plateau Data Center (http://data.tpdc.ac.cn). This dataset demonstrates a high level of predictive accuracy, with a ten-fold cross-validation coefficient of determination (R²) of 0.92 and a root mean square error (RMSE) of 10.76 µg/m³ (16, 17).

#### 2.2.3 Covariates

Previous studies have identified other air pollutants (18), population aging (19), vegetation coverage (20), and socioeconomic development levels (21) as significant factors influencing lung cancer incidence. Consequently, these factors were included as covariates in our analysis. Specifically, PM_10_, NO_2_, and CO were selected to represent other air pollutants. The proportion of the population aged over 60 (Pop>60) was used as a proxy for population aging. The Normalized Difference Vegetation Index (NDVI) served as an indicator of vegetation coverage. Finally, nighttime light data (NTL), the urbanization rate of the resident population (Urbanization), and disposable income per capita (DPI) were employed to represent the level of socioeconomic development.

Data for PM_10_, NO_2_ and CO were obtained from the China High-Resolution High-Quality PM_10_, NO_2_, and CO datasets, respectively, published by the National Tibetan Plateau Data Center (https://doi.org/10.5281/zenodo.3752465; https://doi.org/10.5281/zenodo.4571660; https://doi.org/10.5281/zenodo.4641530) (22–24). Data on the proportion of the population aged over 60 (Pop>60) were sourced from the Tabulation on the 2020 Population Census by County (25). NDVI and nighttime light data (NTL) were derived from the 250-m NDVI dataset for China (https://doi.org/10.11888/Terre.tpdc.300328) and the Long-term NPP-VIIRS-like Nighttime Light Dataset for China (https://doi.org/10.11888/HumanNat.tpdc.302930), respectively, both available from the National Tibetan Plateau Data Center (26). Data on urbanization rate (Urbanization) and disposable income per capita (DPI) were obtained from the Hebei Provincial Statistical Yearbook.

### 2.3 Statistical analysis

#### 2.3.1 Spatial autocorrelation analysis

Spatial autocorrelation analysis can assess the degree of correlation between neighboring elements (27). This study employed a local spatial autocorrelation analysis and used local indicators of spatial association (LISA) to explore the spatial clustering of lung cancer incidence rates in different regions of Hebei Province including high-high, low-low, high-low, and low-high. Areas with high lung cancer incidence are surrounded by other areas with high incidence, which are defined as high-high clusters (low-low, high-low, and low-high definitions are similar) (28). To better detect potential local spatial clustering patterns, the significance level was set at α = 0.1.

#### 2.3.2 Variable selection

To mitigate model overfitting and address multicollinearity, we performed preliminary variable screening using Spearman’s correlation analysis and multicollinearity assessment. The Spearman correlation coefficient between each covariate and PM_2.5_ was calculated, and variables with an absolute correlation coefficient greater than 0.9 were excluded. Subsequently, the remaining variables underwent multicollinearity testing, and only those with a variance inflation factor (VIF) less than 10 were retained for inclusion in the subsequent model.

Based on this criterion, the final set of variables included in the model comprised PM_2.5_, the proportion of population aged over 60 (Pop>60), NDVI, the urbanization rate of the resident population (Urbanization), and disposable income per capita (DPI).

To investigate whether the potential effect of PM_2.5_ on lung cancer incidence varies with regional aging levels or socioeconomic status, interaction terms were incorporated into the model. Specifically, the continuous variables “proportion of population aged over 60” and “disposable income per capita” were converted into binary categorical variables. Using the overall aging level of Hebei Province as a reference, areas with a “proportion of population aged over 60” greater than 20% were classified as the “high aging group,” while the remainder constituted the “low aging group.” Similarly, relative to the average disposable income per capita in Hebei Province, areas with values exceeding 24,000 CNY were classified as the “high income group,” and the remainder as the “low income group.” The subsequent model included both interaction terms (“PM_2.5_ × high aging group” and “PM_2.5_ × high income group”) alongside their respective main effect terms.

#### 2.3.3 Lagged effects of PM_2.5_ on lung cancer incidence

This study employed the cross-correlation function (CCF) to calculate correlations between the annual average PM_2.5_ concentration time series (2008–2022) and the lung cancer incidence time series (2015–2022) in Hebei Province across different lag orders. The lag time corresponding to the maximum absolute cross-correlation coefficient was identified as the optimal lag period for PM_2.5_ effect on lung cancer incidence in subsequent analyses.

#### 2.3.4 Outlier detection

To ensure the robustness of subsequent modeling results and to satisfy the distributional assumptions of parametric models for the dependent variable, this study constructed a regression tree model using the ln-transformed lung cancer incidence rate of each city as the dependent variable and the five core variables identified through prior screening as independent variables. Terminal nodes corresponding to cities with extremely small sample sizes under the two most important variable paths were defined as outliers and excluded from subsequent model analysis.

#### 2.3.5 Regression models

An ordinary least squares (OLS) regression model was first established. OLS is widely used in epidemiological studies (29) and serves as the starting point for spatial regression analysis (30). Its fundamental assumption is that the relationship between independent and dependent variables is spatially stationary. In this study, the ln-transformed lung cancer incidence rate was used as the dependent variable, calculated as follows:


$$\ln(Y_{i})=\beta_{0}+\sum_{k=1}^{p}\beta_{k}X_{ik}+\epsilon_{i}$$

where: $Y_{i}$: Lung cancer incidence rate in the i-th city, $\beta_{0}$: Global intercept, $\beta_{k}$: Global regression coefficient for the k-th independent variable, $X_{ik}$: Value of the k-th independent variable in the i-th city, $\epsilon_{i}$: Random error term.

To facilitate epidemiological interpretation, the coefficients were converted to relative risk (RR) using the formula:


$$RR=\exp\left({\text{β}}_{k}\right)$$

The Geographically Weighted Regression (GWR) model is a local multivariate regression technique that incorporates spatial coordinates into the parameter estimation framework based on OLS. It allows the relationships between independent and dependent variables to vary across geographical locations, thereby effectively capturing spatial non-stationarity in variable relationships (31, 32). The model can be expressed as:


$$\text{ln}\left(Y_{i}\right)={\text{β}}_{0}\left(u_{i},v_{i}\right)+\sum_{k=1}^{p}{\text{β}}_{k}\left(u_{i},v_{i}\right)X_{ik}+{\text{ϵ}}_{i}$$

where: $Y_{i}$: Lung cancer incidence rate in the i-th city, $\left(u_{i},v_{i}\right)$: Spatial coordinates of the i-th city, ${\text{β}}_{0}\left(u_{i},v_{i}\right)$: Local intercept for the i-th city, ${\text{β}}_{k}\left(u_{i},v_{i}\right)$: Local regression coefficient for the k-th independent variable at the i-th city’s location, $X_{ik}$: Value of the k-th independent variable in the i-th city, $\epsilon_{i}$: Random error term.

To facilitate epidemiological interpretation, the coefficients were converted to relative risk (RR) using the formula:


$$RR\left(u_{i},v_{i}\right)\;=\;\text{exp}\left(\beta_{k}\left(u_{i},v_{i}\right)\right)$$

where: $RR\left(u_{i},v_{i}\right)$: Relative risk at the spatial location of the i-th city.

#### 2.3.6 Model evaluation

This study employed the adjusted coefficient of determination (Adjusted R²) and the corrected Akaike Information Criterion (AICc) to evaluate model fit and complexity. A higher Adjusted R² value indicates better model goodness-of-fit, while a lower AICc value reflects a more optimal balance between model parsimony and explanatory power. The model demonstrating both the highest Adjusted R² and the lowest AICc value was selected as the final optimal model.

#### 2.3.7 Statistical software

Data entry and organization of lung cancer case numbers and incidence rates for cities in Hebei Province from 2015 to 2022 were performed using Microsoft Excel. Spatial visualization and map creation were conducted using ArcGIS 10.8. All statistical analyses and modeling were implemented using the Python programming language within the Anaconda 2023 integrated environment. Unless otherwise specified, the significance level (α) was set at 0.05, and a *p*-value of less than 0.05 was considered statistically significant.

## 3 Results

### 3.1 Descriptive analysis

#### 3.1.1 Characteristics of lung cancer incidence in Hebei province

**Figure 1:**
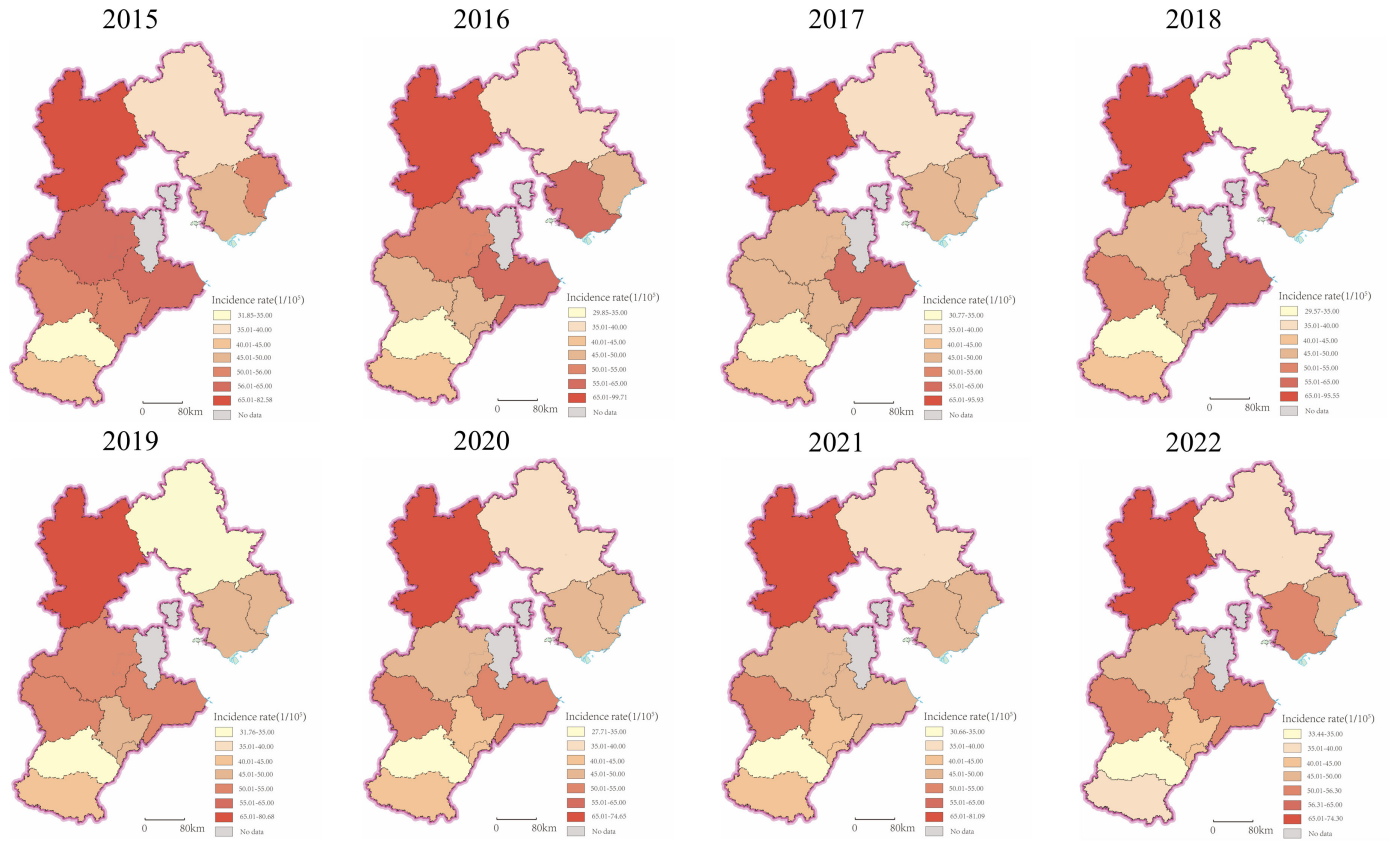
Spatial distribution of lung cancer incidence in Hebei Province, 2015–2022.

From 2015 to 2022, a total of 86,516 new lung cancer cases were reported in the tumor registry areas of Hebei Province, with an average annual incidence rate of 51.15 per 100,000 population over the eight-year period. As shown in **Figure 1**, notable spatial heterogeneity was observed in the distribution of lung cancer incidence across the province. Zhangjiakou recorded the highest incidence rate, while Xingtai exhibited the lowest. The spatial pattern of lung cancer incidence in Hebei Province remained relatively stable throughout the 2015–2022 period.

#### 3.1.2 Distribution of PM_2.5_ in Hebei Province

**Figure 2:**
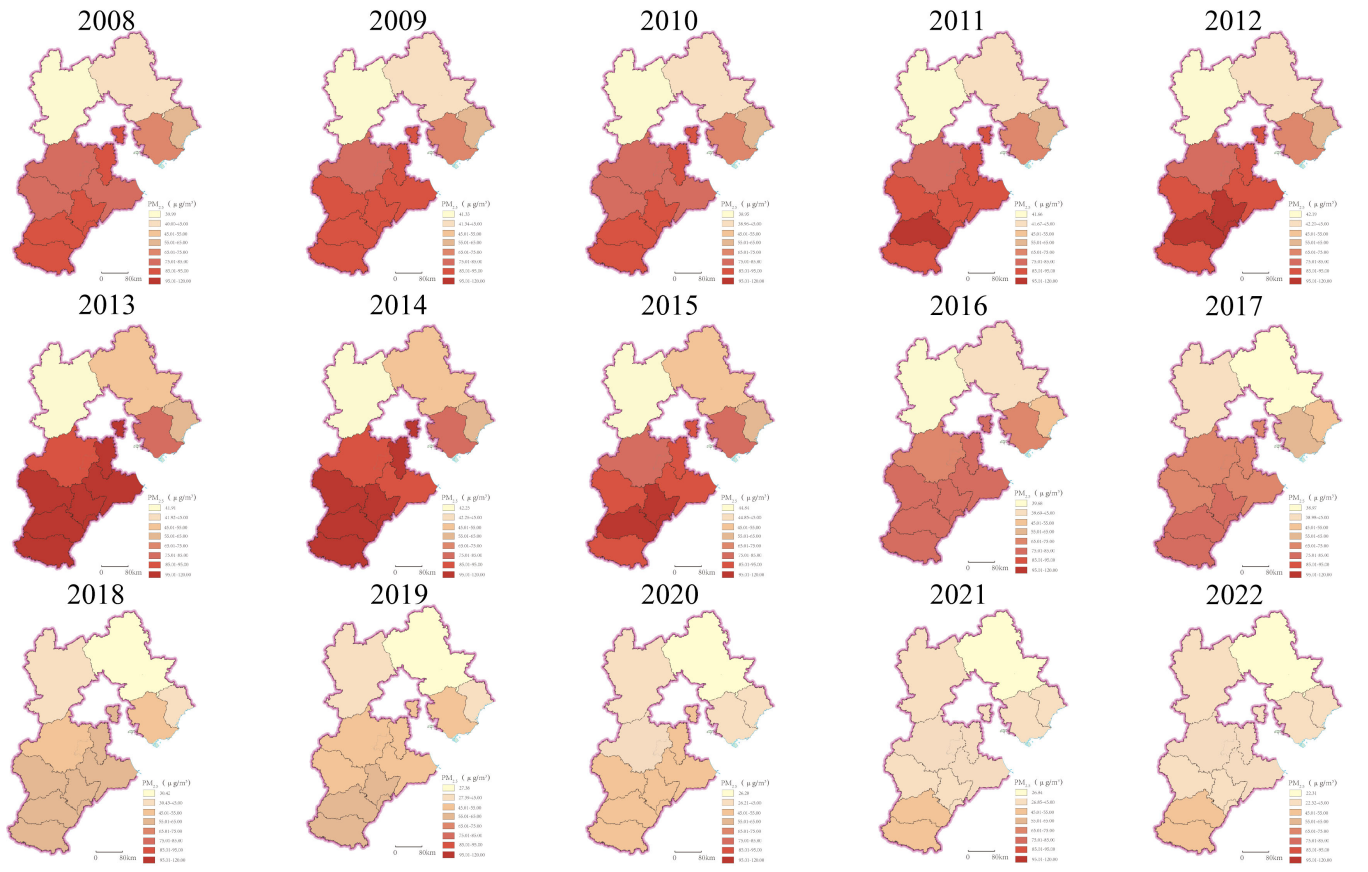
Spatial distribution of PM_2.5_ concentrations in Hebei Province, 2008–2022.

As shown in **Figure 2**, PM_2.5_ pollution in Hebei Province exhibited distinct spatial heterogeneity and temporal trends. During 2008–2022, the spatial distribution of PM_2.5_ concentrations was characterized by a “higher in the south, lower in the north” pattern, while temporally, concentrations showed an initial increase followed by a decline, peaking in 2013 across all regions. Notably, the interregional disparity in PM_2.5_ levels continued to widen from 2008 to 2013, reaching its maximum in 2013 (with a difference of 70.16 μg/m³ between Xingtai, the most polluted, and Zhangjiakou, the least polluted). Thereafter, as PM_2.5_ concentrations decreased, the regional disparity narrowed annually. In 2022, the concentration difference decreased to 25.31 μg/m³, observed between Handan (highest) and Chengde (lowest).

### 3.2 Local spatial autocorrelation analysis

Results from the local spatial autocorrelation analysis (**Table 1**) indicate that the spatial distribution of lung cancer incidence in Hebei Province from 2015 to 2022 was non-random and exhibited significant clustering. The use of traditional global regression models in such a case would fail to account for the inherent spatial heterogeneity of the data. Therefore, it is necessary to incorporate spatial effects in subsequent modeling.

**Table 1:** Results of the local spatial autocorrelation analysis.

City name	2015	2016	2017	2018	2019	2020	2021	2022
Shijiazhuang								
Tangshan								
Qinhuangdao								
Handan		Low-Low				Low-Low		
Xingtai								
Baoding	High-High	Low-High	Low-High	Low-High	High-High	High-High	Low-High	Low-High
Zhangjiakou								
Chengde		Low-High						
Cangzhou								
Hengshui								

### 3.3 Variable selection

**Figure 3:**
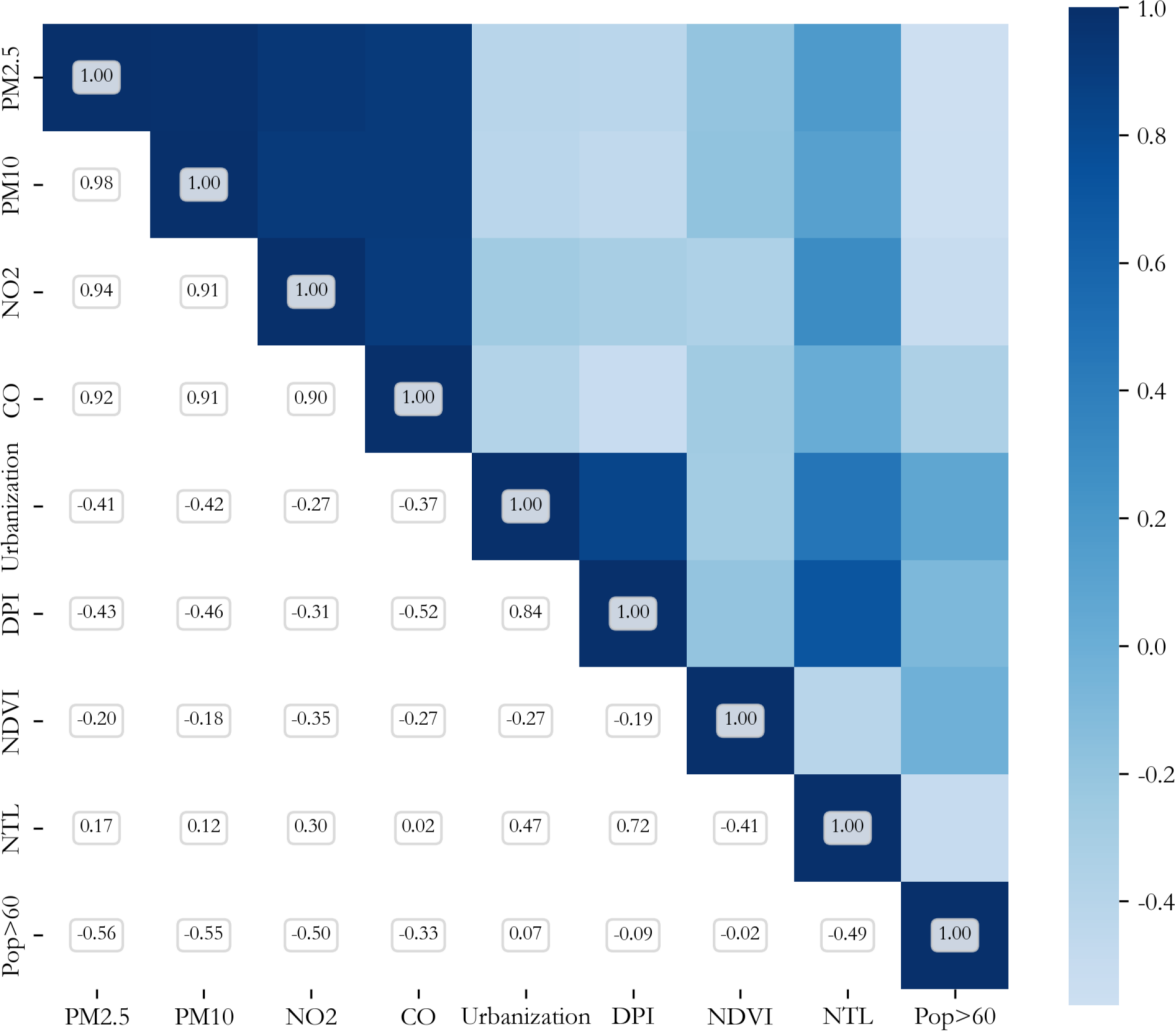
Spearman correlation coefficient heatmap of independent variables.

To mitigate the impact of multicollinearity on model stability, a two-step variable selection procedure was implemented. First, Spearman’s correlation analysis was conducted for preliminary screening. As shown in **Figure 3**, PM_2.5_, PM_10_, NO_2_, and CO exhibited correlation coefficients greater than 0.9, indicating high collinearity. Given that PM_2.5_ was the primary exposure of interest, it was retained while the others were excluded. The remaining variables with correlation coefficients below 0.9—namely PM_2.5_, the proportion of population over 60 years old (Pop>60), NDVI, nighttime light data(NTL), urbanization rate of the resident population (Urbanization), and disposable income per capita (DPI)—were subsequently assessed for multicollinearity. After removing “nighttime light data (NTL)”, all remaining variables had variance inflation factor (VIF) values below 10, indicating acceptable levels of multicollinearity, and were therefore retained for subsequent modeling.

### 3.4 Lagged effects of PM_2.5_ on lung cancer incidence

**Figure 4:**
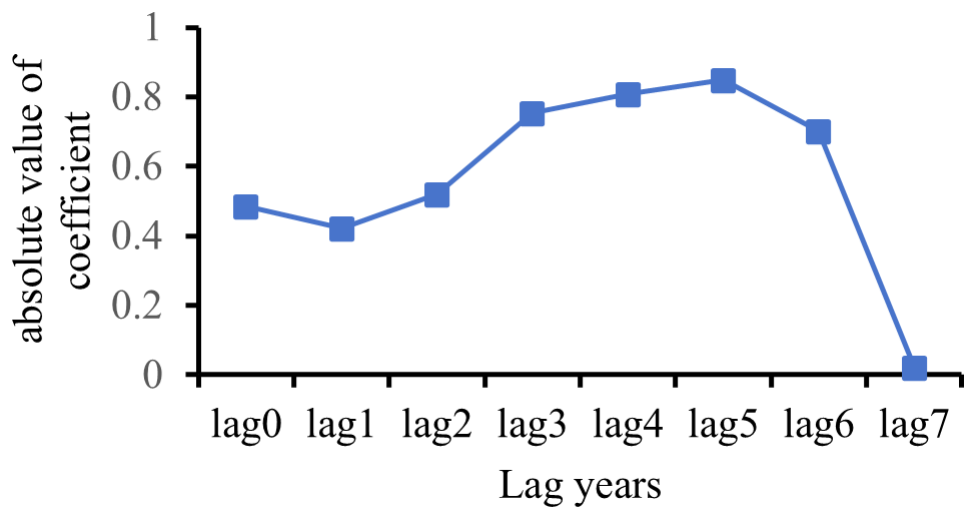
Cross-correlation coefficients between PM_2.5_ and lung cancer incidence at different time lags.

Results from the cross-correlation analysis (**Figure 4**) indicate that the absolute value of the cross-correlation coefficient between PM_2.5_ concentration and lung cancer incidence was greatest at a lag of 5 years. Consequently, the optimal lag period for the effect of PM_2.5_ on lung cancer incidence was determined to be 5 years. Accordingly, the PM_2.5_ concentration with a 5-year lag was adopted as the primary exposure metric in all subsequent models.

### 3.5 Outlier detection

**Figure 5:**
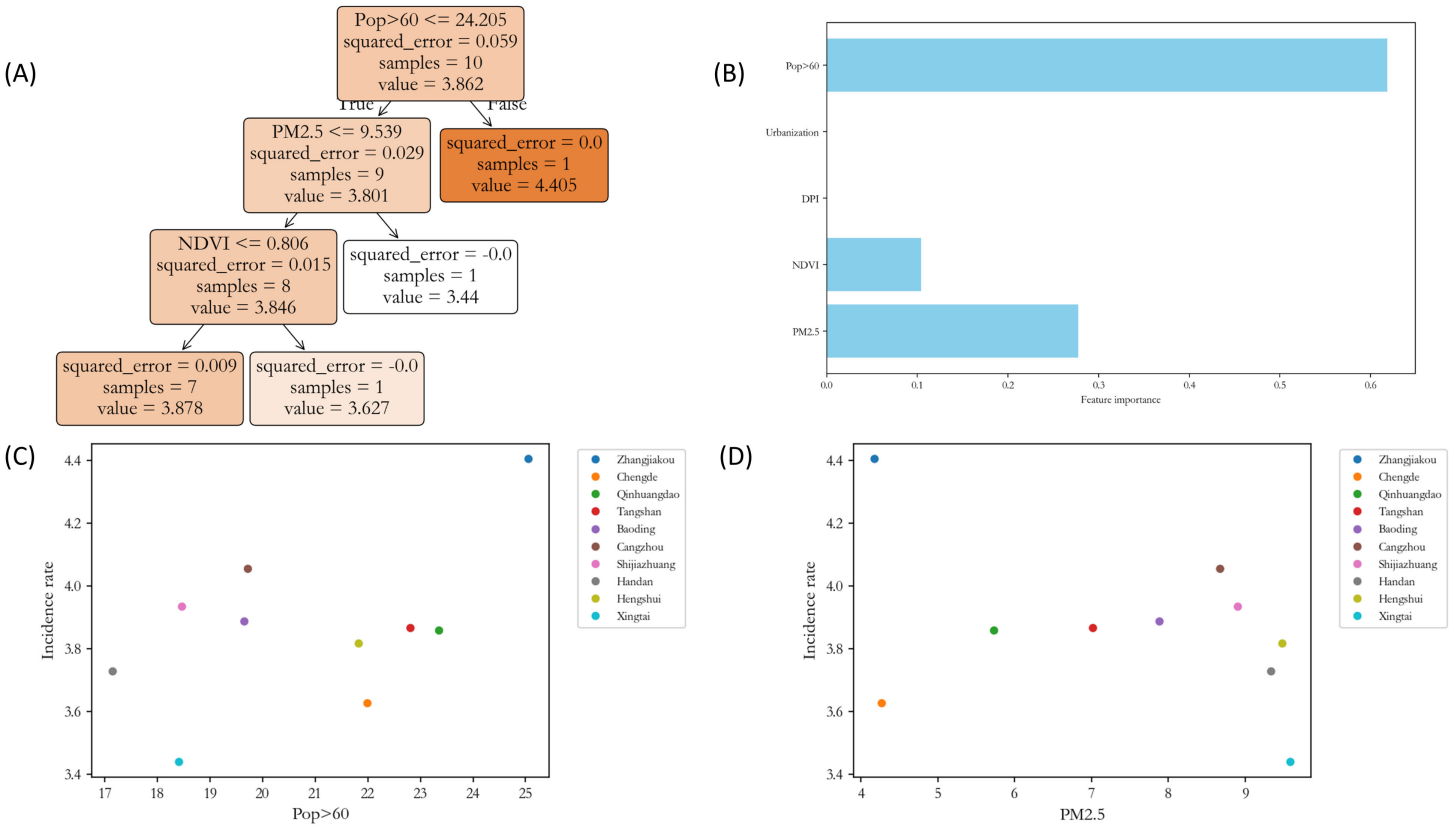
Regression tree analysis and outlier detection for lung cancer incidence. **(A)** Regression tree model structure diagram. **(B)** Feature importance ranking. **(C)** Scatter plot of incidence rate versus elderly population proportion. **(D)** Scatter plot of incidence rate versus PM_2.5_ concentration.

The regression tree and feature importance analysis (**Figure 5**) indicated that the proportion of population over 60 years old (Pop>60) and PM_2.5_ were important factors influencing lung cancer incidence. The decision tree model clearly revealed the hierarchical relationships between these influencing factors and the incidence rate. The analysis identified Zhangjiakou and Xingtai as outlier samples. To ensure the robustness of subsequent models, these two cities were excluded from further analysis.

### 3.6 OLS regression results

The OLS regression results are presented in **Table 2**. PM_2.5_ was identified as a risk factor for lung cancer incidence in Hebei Province, with a relative risk (RR) of 1.079 (95% CI: 1.054-1.104) per 10 μg/m³ increase in concentration. The proportion of the population over 60 years old was also a significant risk factor (RR = 1.109, 95% CI: 1.073-1.147). In contrast, NDVI acted as a protective factor (RR = 0.492, 95% CI: 0.280-0.863). Among the included interaction terms, “PM_2.5_ × High Aging Group” was statistically significant (RR = 0.952, 95% CI: 0.937-0.967). The associations for the remaining variables with lung cancer incidence were not statistically significant. The OLS model demonstrated an Adjusted R² of 0.593 and an AICc of -104.405.

**Table 2:** OLS regression results.

Variable name	Coefficients	Standard errors	t-values	*P*-values	RR and 95%CI
PM_2.5_	0.076	0.011	6.637	<0.001	1.079 (1.054,1.104)
Urbanization	0.006	0.004	1.472	0.146	1.006 (0.997,1.014)
NDVI	-0.710	0.280	-2.530	0.014	0.492 (0.280,0.863)
DPI	-0.427	0.280	-1.531	0.131	0.652 (0.373,1.141)
Pop>60	0.104	0.017	6.208	<0.001	1.109 (1.073,1.147)
PM_2.5_ × high aging group	-0.050	0.008	-6.220	<0.001	0.952 (0.937,0.967)
PM_2.5_ × high income group	-0.001	0.005	-0.145	0.886	0.999 (0.989,1.009)

### 3.7 GWR regression results

**Figure 6:**
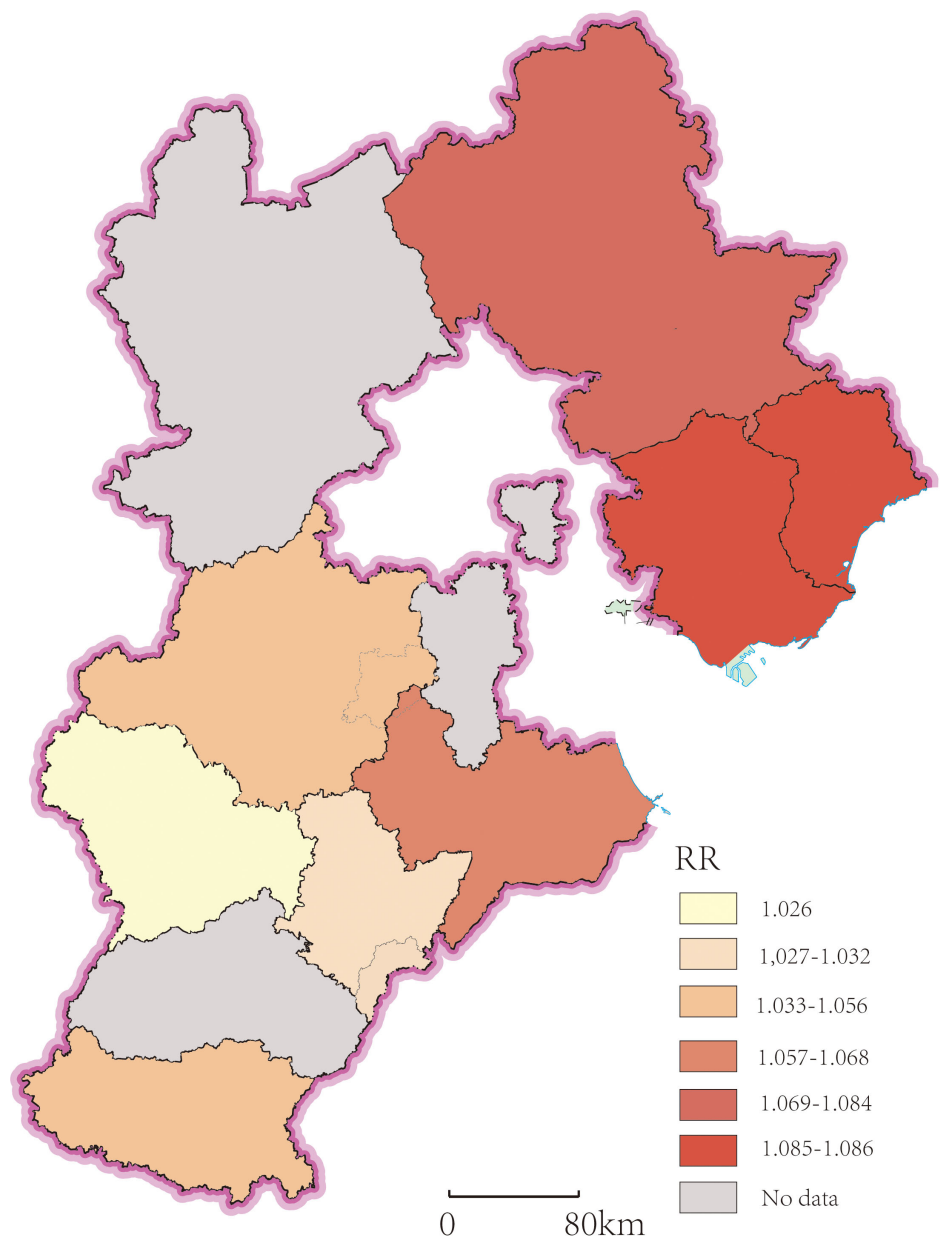
Spatial distribution of PM_2.5_ relative risk (RR).

The GWR model demonstrated an Adjusted R² of 0.681, which was higher than that of the OLS model, and an AICc of -114.601, which was lower than that of the OLS model. These metrics indicate a superior model fit for the GWR model compared to the OLS model. As shown in **Figure 6**, the association between the core variable, PM_2.5_, and lung cancer exhibited significant spatial non-stationarity. The RR for a 10 μg/m³ increase in PM_2.5_ concentration varied spatially between 1.026 and 1.086. Geographically, the strength of the association displayed a pattern of “higher in the north, lower in the south”. The influences of other covariates included in the model on lung cancer incidence also showed varying degrees of spatial heterogeneity, reflecting the geographic complexity of the driving factors behind lung cancer incidence.

## 4 Discussion

This study utilized data from ten cities in Hebei Province and employed both Ordinary Least Squares (OLS) and Geographically Weighted Regression (GWR) models to comprehensively analyze the association of PM_2.5_ on lung cancer incidence and its spatial heterogeneity.

The findings of this study reveal spatial heterogeneity in lung cancer incidence across Hebei Province, with a relatively stable spatial pattern maintained throughout the 2015–2022 period. Results from local spatial autocorrelation analysis confirmed significant spatial clustering of lung cancer incidence, indicating the inadequacy of traditional regression models that ignore spatial effects in this context. This limitation necessitated the adoption of analytical methods capable of accounting for spatial non-stationarity. Consequently, both OLS and GWR models were employed and systematically compared to identify the optimal model for analyzing the association between PM_2.5_ exposure and lung cancer incidence.

For the entire province, each 10 μg/m³ increase in PM_2.5_ concentration was associated with a relative risk (RR) of lung cancer incidence of 1.079 (95% CI: 1.054–1.104). Previous studies have also identified significant associations between PM_2.5_ and lung cancer risk, reporting an RR of 1.18 per 5 μg/m³ increase in Europe (33) and 1.05 per 10 μg/m³ increase in Italy (34). A cohort study in China showed that the 2-year average PM_2.5_ concentration with every 10 μg/m^3^ increase was significantly associated with the incidence of lung cancer (HR = 1.12) (35). We also found that the NDVI was a protective factor for lung cancer incidence (RR: 0.492, 95% CI: 0.280-0.863). This aligns with cohort studies conducted in South Korea (36) and the United Kingdom (37), which also indicated that higher NDVI levels reduce lung cancer risk. Furthermore, an increased proportion of the population over 60 years old was associated with a higher risk of lung cancer. With advancing age, physiological and metabolic processes gradually decline, elevating susceptibility to lung cancer. Additionally, prolonged exposure to risk factors such as smoking and environmental pollution over a lifetime, combined with the cumulative effects of chronic diseases and related complications, further increases the risk of lung cancer in the elderly (38). Regarding the interaction between population aging and PM_2.5_, the high aging group exhibited a lower risk of lung cancer incidence per unit increase in PM_2.5_ compared to the low aging group. This finding, coupled with the regression tree analysis identifying population aging as the most influential factor on lung cancer incidence in this study, leads us to hypothesize that the stronger effect of population aging may have attenuated the measured association of PM_2.5_. This suggests that in an increasingly aged population structure, lung cancer incidence is more likely the outcome of synergistic effects from aging, cumulative environmental exposures, and behavioral factors, wherein the independent contribution of a single environmental factor may become relatively less discernible.

By incorporating spatial dimensions, the GWR model demonstrated superior fit over the OLS model, generating location-specific estimates of the lung cancer risk associated with each 10 μg/m³ increase in PM_2.5_. The results revealed significant spatial non-stationarity in the association between PM_2.5_ and lung cancer. The strength of this association exhibited a “higher in the north, lower in the south” spatial pattern. Intriguingly, this pattern is inversely related to the spatial distribution of PM_2.5_ concentrations across Hebei Province, which generally follows a “lower in the north, higher in the south” gradient. This indicates that the incremental risk of lung cancer incidence per 10 μg/m³ increase in PM_2.5_ is higher in regions with lower baseline pollution levels. This finding is consistent with previous research. A study comprising seven European cohorts reported a non-linear exposure-response relationship between PM_2.5_ exposure and lung cancer mortality, characterized by a steeper increase in risk at lower concentrations and a plateau or attenuated increase at higher concentrations (39). Similarly, a cohort study of non-smokers in China demonstrated a non-linear relationship between PM_2.5_ concentration and lung cancer incidence and mortality, with a steeper slope at lower exposure levels and a flatter slope at higher concentrations (40).

To address the issues caused by particulate matter pollution, China implemented the Air Pollution Prevention and Control Action Plan (APPCAP) from 2013 to 2017. In 2018, the government released the Three-Year Action Plan for Winning the Blue Sky, which proposed concrete measures such as promoting clean heating in northern regions, accelerating the transition from coal to electricity in rural areas, implementing comprehensive treatment of coal-fired boilers, and intensifying the phasing out of small-scale coal-fired boilers (41). These efforts resulted in a significant reduction in the PM_2.5_ concentration in Hebei Province, from 108μg/m³ in 2013 to 36.8μg/m³ in 2022. However, this level remains substantially above the latest World Health Organization (WHO) air quality guideline. Therefore, continued and strengthened control of PM_2.5_ in Hebei Province is still necessary. Developed countries usually implement stricter environmental regulatory policies, maintain better air quality monitoring systems, and have more resources and advanced technologies to reduce pollutant emissions. They use clean energy and improved industrial filtration technologies to effectively control environmental PM_2.5_. We can learn from their successful experiences. For example, the United States has significantly reduced the burden of lung cancer caused by environmental particulate matter pollution by expanding regulatory measures under the Clean Air Act (42). European countries have jointly developed emission reduction technologies and encouraged green enterprises to reduce industrial pollution, thereby lowering health risks (43). Generally, measures such as reducing industrial emissions, promoting vehicles powered by clean energy, expanding public transportation, pursuing healthy urban development, and strengthening the enforcement of national environmental protection policies can all help mitigate the hazards of air pollution. In the long term, there remains significant potential for improvement in areas including industrial and energy structures, lifestyle patterns, urban planning, and regional development strategies.

Lung cancer screening, particularly through the use of low-dose computed tomography (LDCT), is a cost-effective intervention. Early detection of lung cancer through screening has the potential to improve patient survival rates. A study conducted in China demonstrated that lung cancer screening with LDCT can reduce lung cancer mortality by 31.0% (44). In high-risk regions, governments should intensify public awareness campaigns on lung cancer prevention, enhance public knowledge about the disease, and encourage early lung cancer screening to achieve early detection, early diagnosis, and early treatment. These efforts are expected to effectively reduce both the incidence and mortality of lung cancer.

At the same time, Hebei faces many challenges in reducing the burden of lung cancer caused by PM_2.5_. First, the distribution of PM_2.5_ is spatially heterogeneous across Hebei Province, and environmental regulatory policies need to be tailored to local conditions. Second, the proportion of elderly people is increasing year by year, and more targeted measures are needed to alleviate the disease burden of lung cancer caused by aging. Third, there are differences in the level of economic development between different regions. In a national study in China, the association between PM_2.5_ exposure and lung cancer incidence was stronger in regions with lower economic and educational levels (3), and lagging regions face challenges such as economic disparities, insufficient funding for healthcare, limited access to advanced healthcare technologies, lack of awareness of the disease, and insufficient health insurance coverage, among others. Effective cancer management programs need to be developed and cancer prevention and control measures prioritized.

However, this study has several limitations. First, as an ecological study, it provides only population-level estimates of association. Further cohort studies that can link individual health outcomes with PM_2.5_ exposure are needed to verify our findings. Second, we used the average PM_2.5_ concentration of each city as a proxy for exposure, rather than directly measuring the air quality that residents actually breathe. Third, lung cancer incidence is influenced not only by the factors included in this analysis but also by other variables, such as smoking rates, lifestyle habits, education level, and family history of disease. These factors were not incorporated due to difficulties in obtaining relevant data.

## 5 Conclusions

In summary, the association between PM_2.5_ and lung cancer incidence across cities in Hebei Province exhibits spatial heterogeneity. Specifically, for each10μg/m³ increase in PM_2.5_ concentration, the risk of lung cancer incidence is higher in regions with lower baseline pollution levels. This finding suggests that governments and administrative departments, while continuing to prioritize air quality improvement in heavily polluted areas, should also address regions with lower pollution concentrations. Implementing targeted air quality management strategies and healthcare resource allocation policies tailored to local conditions can contribute to sustained improvements in air quality and a reduction in lung cancer incidence. Furthermore, raising public awareness of environmental protection and encouraging necessary precautions against air pollutant exposure are also recommended.

## Data Availability

The raw data supporting the conclusions of this article will be made available by the authors, without undue reservation.
